# Investigation on GaN-Based Membrane Photonic Crystal Surface Emitting Lasers

**DOI:** 10.3390/ma15041479

**Published:** 2022-02-16

**Authors:** Jingtong Bin, Kerui Feng, Wei Shen, Minjia Meng, Qifa Liu

**Affiliations:** College of Telecommunication and Information Engineering, Nanjing University of Posts and Telecommunications, Nanjing 210003, China; b19011829@njupt.edu.cn (J.B.); b19011713@njupt.edu.cn (K.F.); 1220013535@njupt.edu.cn (W.S.); 1221013807@njupt.edu.cn (M.M.)

**Keywords:** GaN-based PCSELs, membrane configuration, confinement factor, gain threshold, field distribution

## Abstract

A GaN-based blue photonic crystal surface emitting laser (PCSEL) featured with membrane configuration was proposed and theoretically investigated. The membrane dimension, photonic crystal (PhC) material, lattice constant and thickness were studied by RCWA (Rigorous Coupled Wave Analysis), FDTD (Finite Difference Time Domain) simulations with the confinement factor and gain threshold as indicators. The membrane PCSEL’s confinement factor of active media is of 13~14% which is attributed to multi-pairs of quantum wells and efficient confinement of the mode in the membrane cavity with air claddings. The excellent confinement factor and larger Q factor of resonance mutually contribute to the lower gain threshold of the design (below 400 cm^−1^ for GaN-PhC with 100 nm thick top and bottom GaN layer, 40 nm hole radius and 40 nm depth). The PhC confinement factor exceeds 13% and 6% for TiO_2_-PhC with 80 nm and 60 nm PhC thickness and 20 nm and 40 nm distance between PhC and active media, respectively. It is around two times larger than that of GaN-PhC, which is attributed to the higher refractive index of TiO_2_ that pulls field distribution to the PhC layer.

## 1. Introduction

Currently, GaN-based blue lasers play a critical role in optical communications, especially underwater communication [1,2]. Besides, based on the outstanding properties of GaN laser diodes [3,4,5,6], they have been widely used in high-density data storage, laser display, material processing and biomedical devices.

The first semiconductor photonic crystal surface emitting laser (PCSEL) was realized in an InP-based system in 1999 [7]. GaN-based both optically and electrically pumped PCSELs were demonstrated initially in 2008 [8,9]. PCSELs are candidates to operate as a high-power source with a single mode and narrow divergence angle lasing over a broad area [10,11,12,13,14,15]. Compared with vertical cavity surface-emitting lasers (VCSELs), it does not need the highly reflective Bragg mirror, which is very difficult to implement in a GaN-based material system [16].

Photonic crystals (PhCs) are an excellent structure to form cavities with ultra-high Q-factors and ultra-small mode volumes denoted as high Q/V_m_, which are widely reported [17,18,19,20]. These are PhC defect mode cavities with a small cavity region by using surrounding PhCs as the reflector. Lasers based on such nanocavities can achieve strong Purcell effect and low-gain threshold, but a finite output power and difficult electrical-pump configuration. PCSELs utilize the multidirectional distributed feedback (DFB) effect of two-dimensional perfect PhCs. The DFB induces in-plane coupling and diffract perpendicularly by the first-order Bragg diffraction of PhCs. In PCSELs, the mode coupling in PhCs and gain media are both critical, where the one provides mode oscillating and extracts light vertically, the other achieves oscillation gain.

In GaN-based PCSELs, PhCs formed by etching through the gain media are the most commonly reported method [10,21,22,23] because they can obtain high coupling strength both in PhCs and gain media. However, it damages the active material, which affects the quantum wells’ luminescence and the uniform distribution of the injection current. This is the reason why these kinds of structures are always suitable to be optically pumped. Moreover, the dry-etch through quantum wells introduces defects that ultimately reduce the IQE of the device [24]. On the other hand, in order to realize high field coupling in PhCs and quantum wells, all reported GaN-based PCSELs have to employ a relatively thick AlGaN cladding layer to form a waveguide-like structure. The p-doped AlGaN has extremely low electrical conductivity, which results in high operating energy costs [25,26]. Despite the use of a thick AlGaN cladding layer, confinement factors in PhCs and the active region of reported electrically pumped GaN-PCSELs are still in a low value range [8,27].

In this work, GaN membrane blue PCSEL is theoretically designed based on a particular fabrication process and material platform. A GaN membrane embedded with multi-quantum wells (MQWs) featuring controllable thickness by III–V; compound dry etching can be realized. The membrane forms a waveguide-like structure with air as the top and bottom claddings. This design avoids the necessity of an AlGaN layer and possesses high coupling strength in PhCs and MQWs because the high refractive index contrast between GaN and air can confine fundamental modes efficiently. Based on this air cladding cavity, the confinement factors (coupling strength) in PhCs and MQWs are considerable, which makes it feasible to use shallow-etched surface PhCs. Compared with the structure of buried PhCs inside the GaN layer [8,27], the PhCs etched on the GaN top surface are easier to fabricate. The regrowth step in buried PhC fabrication has to put the device back into the growth chamber after etching, suffering from poor interfaces and bringing high interface recombination [28]. This kind of non-radiative recombination will damage the laser properties. Besides, the surface PhCs’ design is more flexible and optimistic to obtain high coupling strength, especially in PhCs, thus realizing good performance of the laser. Here, we fully explore the PhCs and membrane parameters, optimize the confinement factors and gain threshold by tailoring the fundamental guided modes in the membrane. Square lattice PhCs based on GaN and TiO_2_ are investigated and compared.

## 2. Materials and Methods

**Figure 1:**
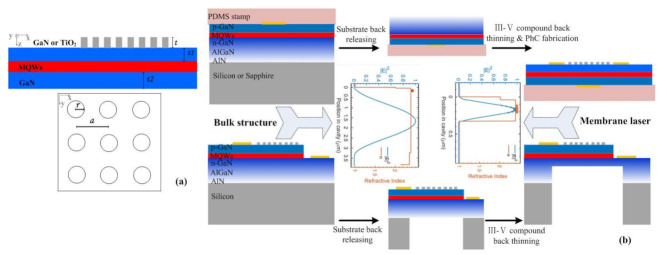
(**a**) Schematics of the proposed GaN membrane PCSEL structures and (**b**) the fabrication process. The figure denotes the bulk wafer structure and the calculated fundamental mode distribution with *t*_1_ = *t*_2_ = 100 nm.

The schematic of the proposed design and fabrication process is shown in Figure 1. The laser structure is composed of square lattice TiO_2_ or GaN PhC layer with thickness *t*, lattice constant *a*, hole radius *r*, under PhC GaN layer with thickness *t_*1*_*, bottom GaN layer with thickness *t_*2*_*, 9 pairs of InGaN 3 nm / GaN 10 nm MQWs layer. The fabrication was started with GaN-on-silicon or GaN-on-sapphire wafers, whose structure consists of silicon or sapphire substrate, ~1000 nm AlN and AlGaN buffer layer, and ~3000 nm n-GaN. Two kinds of double-side processes were developed to obtain GaN membrane PCSEL. Route I: First, the wafer with p-contact was bonded with the PDMS stamp; then, the silicon or sapphire substrate was released by dry etching or laser lift-off; finally, GaN thinning, PhC and n-contact fabrication were performed. Route II: PhC layer was fabricated first on top of p-GaN; then p-mesa and p,n-contact were fabricated; backside substrate releasing and AlN-AlGaN-GaN etching was performed to realize a specific thick membrane cavity. The freestanding GaN-based PCSELs feature mode tuning by GaN-thinning tailoring the guided mode to realize high confinement factors. From a comparison of the electric field distribution of the fundamental guided mode in Figure 1, the high confinement factors in MQWs and PhCs could be obtained in the membrane structure due to the high refractive contrast between GaN and air. The field distribution and confinement factor were calculated by the Stanford Stratified Structure Solver (S4) based on Rigorous Coupled Wave Analysis (RCWA). S4 is a frequency domain code used to solve layered periodic structures. Internally, it is also called the Fourier Modal Method and the S-matrix algorithm. It allows for fast, accurate prediction of optical propagation through complex 3D structures. A transverse electric (TE) wave was considered in the simulation.

We performed systematic simulations on structure parameters to investigate the influence on laser properties. The confinement factor in PhCs and gain threshold at resonant wavelengths were calculated and taken as the indicators. The gain threshold was derived from Equation (1):

(1) $$g_{th}\;=\;\frac{\alpha }{\Gamma_{QW}}$$

where *α* is vertical radiation loss, *Γ_QW_* is the field confinement factor in QWs. For large PhC areas, such as those larger than 400 μm in length, the in-plane loss and internal loss can be negligible [12,28,29,30]. So, only the vertical radiation loss was considered here and calculated by Equation (2):

(2) $$\alpha \;=\;\frac{2\pi }{Q\;\cdot \;a}$$

where *a* is the lattice constant and *Q* is the quality factor obtained by Fano fitting the guided resonances in reflection spectra. The confinement factor in MQWs and PhCs can be calculated by Equation (3):

(3) $$\Gamma_{QW}\;=\;\frac{\int_{QW}(E_{x}^{2}\;+\;E_{y}^{2})\;\cdot \;dv}{\int_{cav}(E_{x}^{2}\;+\;E_{y}^{2})\;\cdot \;dv}\Gamma_{PhC}\;=\;\frac{\int_{PhC}(E_{x}^{2}\;+\;E_{y}^{2})\;\cdot \;dv}{\int_{cav}(E_{x}^{2}\;+\;E_{y}^{2})\;\cdot \;dv}$$

The confinement factor was calculated by extracting the *E_x_* and *E_y_* components of the electric field, as the DFB mainly used the oscillation on the PhC surface plane. In S4 simulations, the refractive indexes are set as follows: *n_air_* = 1, *n_silicon_* = 4.68, *n_GaN_* = 2.4, *n_InGaN_* = 2.46, *n_AlN_* = 2.15, *n_AlGaN_* = 2.354. The thickness of the substrate and the above air are set to infinite.

## 3. Results

**Figure 2:**
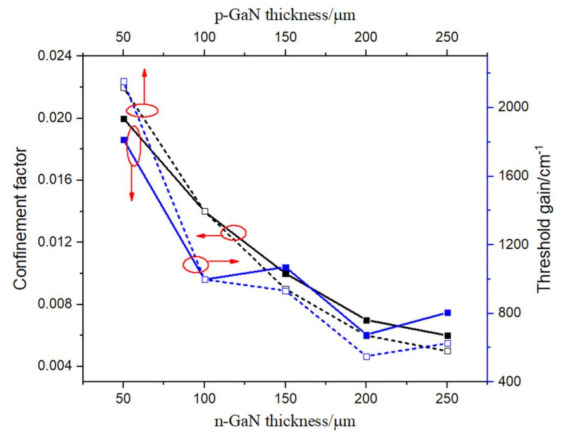
Confinement factor in PhCs and threshold gain plots as functions of n-GaN and p-GaN thickness, with holes depth 40 nm, lattice constant 194 nm, and hole radius 60 nm.

Figure 2 shows the investigation results of p-GaN and n-GaN thickness *t*_1_ and *t*_2_, while the top surface GaN PhC hole depth *t* is 40 nm, square lattice constant *a* is 194 nm, and hole radius *r* is 60 nm. Both the confinement factor and threshold gain decrease as GaN thickness increases. Top GaN and bottom GaN have similar influences on the laser cavity properties. The thinner the top and bottom GaN, the higher the field intensity and the smaller the distance between the active layer and the PhC layer, which produce larger confinement factors. However, the quality factors (Q value) deteriorate along with the membrane thickness, becoming smaller. From Equations (1) and (2), the gain threshold relates to both the Q value and *Γ*_QW_. In this case, the Q factor dominates the influence on *g_th_*, thus bringing on larger *g_th_* at smaller membrane thickness. Therefore, for the membrane thickness, it is a trade-off to both consider higher PhC coupling strength and lower gain threshold. The membrane thickness should be thin enough to get enough field coupling strength in PhC, which is the necessity of lasing, but should not be too thin to hold a relatively lower threshold.

**Figure 3:**
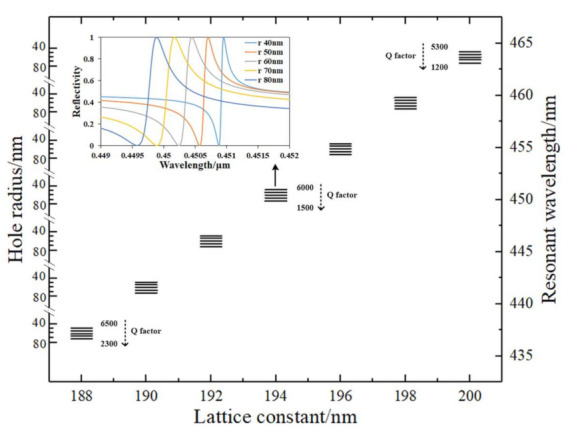
Influence of lattice constant and hole radius on resonant wavelength and mode Q factor.

Square lattice photonic crystals formed by top GaN partially etched (40 nm depth) with different lattice constants and hole radii were investigated. The n-GaN and p-GaN thicknesses were fixed at 100 nm. The resonant frequency can be tuned by the PhC lattice constant. Considering the luminous band 430~470 nm of the InGaN/GaN MQWs, we designed the lattice constant in the range of 188 to 200 nm. The resonance wavelength is linearly proportioned to the lattice constant with the tune rate of *λ_peak_/lattice constant* = ~2.3 nm/nm. The results are exhibited in Figure 3. At each lattice constant, a 40~80 nm hole radius was calculated. We focus on this hole radius range based on the fabrication realizability and the resonance properties. On the one hand, a smaller hole radius is beneficial for a lower gain threshold and larger confinement factors. On the other hand, the fabricated hole radius is still larger than 40 nm based on the traditional fabrication process. The results in Figure 3 denote that the lattice hole radius has a relatively fine tuning effect on the resonant wavelength. More importantly, the Q factor of the resonance is dramatically influenced by the hole radius. A smaller hole possesses a sharp reflectance peak, which represents a larger Q factor. The reflectance curve at a different hole radius of the 194 nm lattice constant is exhibited as the inset in Figure 3. Therefore, a smaller hole radius can realize less vertical loss, which benefits obtaining a lower threshold gain of the laser. Some of the Q factors are denoted in the figure, from which we can know that the smaller wavelength resonance mode at a smaller lattice constant has slightly higher Q factors.

We also investigated TiO_2_ as PhCs materials here to give a comparable design for the membrane GaN PCSELs. This reflects the advantage of flexible design for the proposed surface PhCs. TiO_2_ has negligible absorption at a ~450 nm wavelength with a 2.55 refractive index [30,31,32]. This high refractive index will pull field distribution move to PhCs and thus obtain a high PhC confinement factor. Besides, TiO_2_ is a widely available material with compatible growth properties and can be easily fabricated as micro-nano structures, such as gratings and photonic crystals [31,32,33,34,35].

Different lattice constants and hole radius designs have the function of realizing different wavelength mode lasing, as illustrated in Figure 3. Apparently, every mode has different laser performance as the indicators listed in Table 1. Parameters and performances for TiO_2_-PhC lasers are also listed in Table 1. Other parameters were set the same as GaN-PhC, with a 40 nm PhC layer thickness, 60 nm GaN layer under PhC and 100 nm bottom GaN layer. From the table, smaller holes exert positive effects on both PhC confinement factors and gain threshold. As shown in Table 1, smaller holes can support resonance with smaller radiation loss, thus reducing the threshold of the laser. We also note that the threshold corresponds to the confinement factor in MQWs from Equation (1). In all of our calculated results, *Γ_QW_* are in the range of 13~14% at various parameters, which has a finite influence on threshold changes. However, it should be pointed out that the larger *Γ_QW_* contributes to the relatively lower gain threshold. The larger *Γ_QW_* is due to multi-pairs of quantum wells and the good confinement of the mode in the membrane cavity with air claddings. TiO_2_-PhC has a larger PhC confinement factor and a higher threshold compared to GaN-PhC. The strong coupling strength is attributed to the high refractive index of TiO_2_, which pulls the field distribution to the surface. Smaller holes have larger PhC confinement factors, which is also due to the higher effective refractive index of the PhC layer.

**Figure 4:**
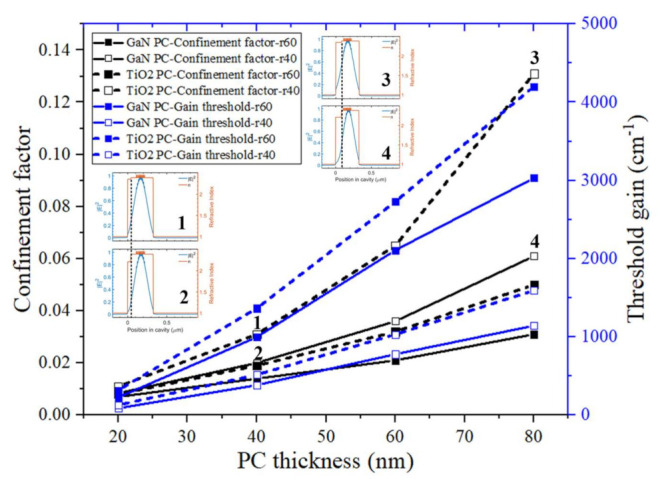
PhC confinement factor and gain threshold changing with PhC layer thickness of GaN-PhC and TiO_2_-PhC at hole radius of 40 nm and 60 nm, respectively. The insets exhibit mode distribution in layers at 40 nm hole radius with (**1**) 40 nm and (**3**) 80 nm TiO_2_-PhC hole depth and (**2**) 40 nm and (**4**) 80 nm GaN-PhC hole depth.

Hole depth (PhC layer thickness) also has obvious influences on PCSELs. Figure 4 exhibits the indicators changing with PhC hole depth while keeping the distance from active region to top surface at 100 nm, lattice constant at 194 nm and bottom GaN thickness at 100 nm. The confinement factors and gain threshold both increase as the hole depth increases, no matter what the PhC material or hole radius is. Along with etching deeper, the distance between PhC and active region decreases, achieving higher field coupling strength in PhC. However, the radiation loss of the resonant mode increases when holes become deeper, which causes the threshold to increase. The comparison between GaN-PhC and TiO_2_-PhC is clearly shown in Figure 4. PCSELs with TiO_2_-PhC possess a larger PhC confinement factor and larger threshold gain. The TiO_2_-PhC confinement factor is around two times larger than that of GaN-PhC. The inset shows the field distribution of GaN-PhC and TiO_2_-PhC at a 40 nm hole radius with 40 nm and 80 nm hole depth, respectively. Obviously, in both situations, the fundamental field profile moves to the PhC layer when changing GaN PhC to TiO_2_ PhC. Up to 13% confinement factor can be achieved with 80 nm hole depth.

**Figure 5:**
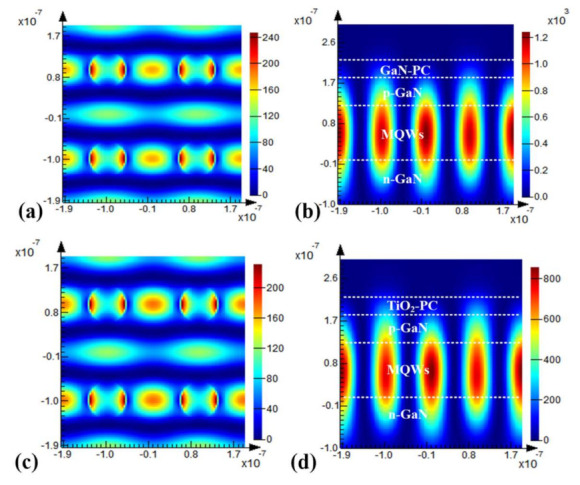
Field distribution in PhC surface plane (**a**,**c**) and cross-section plane (**b**,**d**) of the PCSELs with lattice constant 194 nm, hole radius 40 nm, hole depth 40 nm of GaN-PhC (**a**,**b**) and TiO_2_-PhC (**c**,**d**) at resonant peaks of 450.95 nm and 451.67 nm, respectively.

The finite difference time domain (FDTD) method was employed to calculate the field distribution. Figure 5a,b exhibit field distribution in the PhC layer and cross-sectional view of the PCSELs with lattice constant 194 nm, hole radius 40 nm, and hole depth 40 nm of GaN-PhC at resonant peaks of 450.95 nm. Figure 5c,d exhibit field distribution in the PhC layer and cross-sectional view of the PCSELs that of TiO_2_-PhC at resonant peaks of 451.67 nm. Resonances of the fundamental mode at these two wavelengths are apparently formed and oscillating confined in the membrane. Both fields have large coupling in MQWs and small coupling in the PhC layer, and the coupling in TiO_2_-PhC is slightly bigger than that in GaN-PhC. The FDTD simulation results verify the preceding results from RCWA.

**Figure 6:**
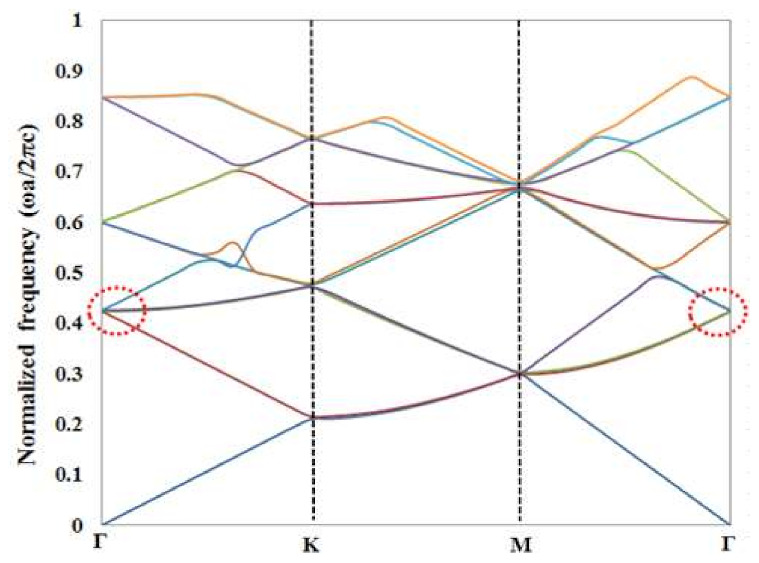
Photonic band structure of the proposed GaN-PCSELs with TiO_2_ PhC of TE polarization.

The photonic band structure of the proposed GaN-PCSELs with top TiO_2_ PhC was calculated by MIT photonic bands (MPB), based on the fully vectorial plane wave expansion method (PWE). The general calculation approach can be referred to in many reports [36,37]. A lasing oscillation is considered to occur at the band edges of the *Γ* point, which is denoted in the figure. From Figure 6, the normalized frequency of the band edge in the band structure is around 0.42. Based on this, the calculated lattice constant is around 190 nm. This is in accordance with the designed parameters above, which explains and verifies the design. The GaN membrane PCSEL design based on the band-edge effect has also been verified by photonic band structure calculation in our previous article [38].

## 4. Conclusions

In this work, a membrane structure was proposed in PCSELs for GaN-based blue lasers. The slab-waveguide-like membrane gives birth to both large confinement factors in PhCs and MQWs, which is a promising candidate to be a distinguished PCSEL cavity. Parameters such as membrane thickness, PhC lattice constant, hole radius and depth, as well as different PhC materials were investigated by RCWA simulation to analyze the influence on confinement factors and gain threshold.

The membrane thickness should be thin to get enough field coupling strength in PhC but should not be too thin to hold a relatively lower threshold. A smaller hole radius is preferred because it features higher coupling strength in PhCs and less vertical loss, which benefits obtaining a lower gain threshold. The lattice constant is linearly proportioned to the resonance wavelength and has a tune rate of *λ_peak_/lattice constant* = ~2.3 nm/nm. The confinement factors and gain threshold both increase with hole depth. TiO_2_-PhC brings a higher PhC confinement factor and higher threshold compared to GaN-PhC. The TiO_2_-PhC confinement factor is around two times larger than that of GaN-PhC. This design and investigation paves the way for near future experimental investigations and realization of GaN-based membrane blue PCSELs.

On the other hand, there are some challenges for the realization and application of these membrane lasers. One is that the membrane configuration may suffer a high thermal resistance problem in application. The other is the efficient current injection, which is a problem for GaN-based PCSELs, especially for the membrane structure. In this work, we proposed two ways to realize the laser in Figure 1 with n-GaN and p-GaN on the top, respectively. The former may suffer from high power absorption in PDMS, while the latter is constricted to low carrier mobility of p-GaN.

## Figures and Tables

**Table 1:** Specific list of *Γ*_PhC_, g_th_, at different GaN-PhC and TiO_2_-PhC parameters (*t* = 40 nm, *t*_1_ = 60 nm, *t*_2_ = 100 nm. *a* and *r* unit in *nm*, *g_th_* unit in cm^−1^).

	Lattice Constant (*a*)	GaN-PhC			TiO_2_-PhC		
Hole Radius (*r*)		188	194	200	188	194	200
40		*Γ_PhC_* = 2% *g_th_* = 376	*Γ_PhC_* = 2% *g_th_* = 379	*Γ_PhC_* = 2.1% *g_th_* = 409	*Γ_PhC_* = 3% *g_th_* = 556	*Γ_PhC_* = 3.1% *g_th_* = 516	*Γ_PhC_* = 3.1% *g_th_* = 571
50		*Γ_PhC_* = 1.6% *g_th_* = 613	*Γ_PhC_* = 1.7% *g_th_* = 653	*Γ_PhC_* = 1.7% *g_th_* = 700	*Γ_PhC_* = 2.4% *g_th_* = 904	*Γ_PhC_* = 2.5% *g_th_* = 922	*Γ_PhC_* = 2.6% *g_th_* = 945
60		*Γ_PhC_* = 1.3% *g_th_* = 941	*Γ_PhC_* = 1.4% *g_th_* = 998	*Γ_PhC_* = 1.4% *g_th_* = 1113	*Γ_PhC_* = 1.8% *g_th_* = 1347	*Γ_PhC_* = 1.9% *g_th_* = 1365	*Γ_PhC_* = 2% *g_th_* = 1503
70		*Γ_PhC_* = 1% *g_th_* = 1155	*Γ_PhC_* = 1% *g_th_* = 1339	*Γ_PhC_* = 1.1% *g_th_* = 1475	*Γ_PhC_* = 1.2% *g_th_* = 1661	*Γ_PhC_* = 1.4% *g_th_* = 1846	*Γ_PhC_* = 1.5% *g_th_* = 2055
80		*Γ_PhC_* = 0.7% *g_th_* = 1046	*Γ_PhC_* = 0.8% *g_th_* = 1429	*Γ_PhC_* = 0.9% *g_th_* = 1707	*Γ_PhC_* = 0.9% *g_th_* = 1541	*Γ_PhC_* = 1% *g_th_* = 1934	*Γ_PhC_* = 1.1% *g_th_* = 2305

## Data Availability

Not applicable.
